# A comparison of seasonal flexibility in pectoralis muscle fiber type and enzyme activity in migratory and resident sparrow species

**DOI:** 10.1242/jeb.249392

**Published:** 2025-02-04

**Authors:** Louisa M. Lewicki, Marina Zhang, James F. Staples, Christopher G. Guglielmo, Catherine M. Ivy

**Affiliations:** ^1^ Department of Biology, Western University, London, Ontario, Canada, N6A 3K7; ^2^ Centre for Animals on the Move, Advanced Facility for Avian Research, Department of Biology, Western University, London, Ontario, Canada, N6A 3K7; ^3^ Department of Biology, University of Saskatchewan, Saskatoon, Saskatchewan, Canada, S7N 5C8

**Keywords:** Myosin ATPase activity, Capillary staining, Songbird

## Abstract

The pectoralis muscle in birds is important for flight and thermogenesis. In migratory songbirds this muscle exhibits seasonal flexibility in size, but whether this flexibility reflects changes in muscle fiber type has not been well documented. We investigated how seasonal changes in photoperiod affected pectoralis muscle fiber type and metabolic enzymes, comparing among three closely related sparrow species: two seasonal migrants and one year-round, temperate climate resident. We quantified fast oxidative glycolytic (FOG) and fast glycolytic (FG) fibers histologically, and measured activities of citrate synthase (CS) and lactate dehydrogenase (LDH) in the pectoralis muscle of the three species that were acclimated to long or short periods of daylight. In all species, FOG was the predominant fiber type, but song sparrows had FG fibers regardless of daylight conditions. By contrast, Lincoln's sparrows incorporated FG fibers only under short-daylight conditions, and house sparrows did not significantly express FG fibers, regardless of daylight length. Both migratory species increased LDH activity in short-daylight conditions but did not alter CS activity. In contrast, resident house sparrows did not alter CS or LDH activity with changes in daylight length. Our findings suggest that the presence of FG fibers is important for seasonal flexibility in LDH activity. Additionally, migratory species exhibited seasonal flexibility in muscle fiber type and enzyme activity, presumably to support migratory flight, while the resident species did not exhibit such seasonal flexibility, suggesting that this consistent phenotype is important year-round, despite changing thermogenic requirements.

## Teaser

**Summary:** Migratory sparrows exhibit seasonal flexibility in lactate dehydrogenase activity, but not citrate synthase activity, which may be associated with the presence of fast glycolytic muscle fibers in the non-migratory season.

## INTRODUCTION

The pectoralis muscle constitutes 10–25% of a bird’s body mass and plays an important role in flight and thermoregulation (Greenewalt, 1962; Lundgren and Kiessling, 1988). This muscle generates force for the downstroke of the wing during flight and is continuously used for many hours to days during migratory flight (Butler, 1991; Goldspink, 1980). The pectoralis muscle therefore requires large amounts of oxygen to sustain flight, making migratory flight one of the most aerobically demanding forms of migration (Butler, 1991). For many resident (non-migratory) songbird species and those that experience colder climes, the pectoralis muscle is used for flying short distances within the home range, but is particularly important for thermoregulation in cold seasons, producing endogenous heat through shivering (Jimenez, 2020; Marsh and Dawson, 1989). This dual role of the pectoralis highlights its importance in songbird physiology, but how muscle morphology and biochemistry changes seasonally and/or between resident and migratory songbird species is only just starting to be understood (Chang et al., 2024; Coulson et al., 2024; Dick, 2017; DuBay et al., 2020; Ivy and Guglielmo, 2023; Lundgren and Kiessling, 1988).

The muscle fiber types constituting the pectoralis vary among bird species, largely reflecting their performance capacities. Slow oxidative (SO) fibers, which have high aerobic enzyme activities (Fitts et al., 1989), are typically observed in the pectoralis muscle of soaring or flightless birds (Goldspink, 1980; Rosser and George, 1986; Welch and Altshuler, 2009). These fibers resist fatigue and are used for slow, repetitive actions, but they produce little force (Goldspink, 1980; Rosser and George, 1986; Welch and Altshuler, 2009). Fast oxidative glycolytic (FOG) fibers also resist fatigue and rely on aerobic metabolism to maintain high contraction frequency for long durations (Fitts et al., 1989; Peter et al., 1972). These fibers are common in all bird pectoralis muscles and are assumed to form most of the flight muscle fibers in smaller (<20 g) songbirds, as they can sufficiently fulfill many aspects of bird flight (e.g. burst responses, prolonged flight) (Torrella et al., 1998; Welch and Altshuler, 2009). In contrast, fast glycolytic (FG) fibers rely on anaerobic enzyme activity and produce large amounts of force for short durations but they lack endurance (Fitts et al., 1989; Welch and Altshuler, 2009). This muscle fiber type is used by birds during burst flight or escape maneuvers that require sudden or rapid acceleration (Parker and George, 1975). FG fibers are commonly observed in the pectoralis of larger birds (>30 g), although some small songbird species (<20 g) have a mix of FOG and FG fibers present in their pectoralis (Chang et al., 2024; DuBay et al., 2020; Lundgren and Kiessling, 1988; Qu et al., 2020; Rosser and George, 1986). These findings suggest that other small songbirds may have FG fibers present, but further research is required.

Pectoralis muscle mass exhibits reversible flexibility in response to changing seasonal conditions (Bauchinger and Biebach, 2005; Jimenez, 2020; Le Pogam et al., 2020; Piersma et al., 1999), but whether this is associated with changes in muscle fiber type and/or fiber transverse surface area has not been well documented. In some songbirds, pectoralis muscles exhibit seasonal changes in fiber type, fiber transverse surface area (Chang et al., 2024; Ivy and Guglielmo, 2023; Lundgren and Kiessling, 1988) and capillary density (Mathieu-Costello et al., 1998), although these changes appear to depend on species or, in some cases, individuals within a species (Chang et al., 2024; Ivy and Guglielmo, 2023). Additionally, some of these responses may depend on migratory distance and/or whether a bird is a migratory or resident species (Chang et al., 2024; Lundgren and Kiessling, 1988), but this has not been well investigated in the literature.

Metabolic enzyme activities in the pectoralis muscle also exhibit seasonal flexibility (Coulson et al., 2024; Dick, 2017; Swanson et al., 2020), which could result from changes in fiber type or flexibility of enzyme activities within a fiber type (Fitts et al., 1989). For example, citrate synthase (CS), which plays a crucial role in aerobic metabolism to produce ATP, increases in activity during migratory periods in small songbirds (Coulson et al., 2024; Dick, 2017; Lundgren and Kiessling, 1985, 1986). By contrast, resident songbirds exhibit no change with CS activity between summer and winter conditions (Marsh and Dawson, 1989; Swanson et al., 2020; Yacoe and Dawson, 1983). Similarly, lactate dehydrogenase (LDH), which is involved in anaerobic metabolism and converts pyruvate to lactate when oxygen is limited, exhibits plasticity, with reduced activity during migratory periods (Coulson et al., 2024; Dick, 2017; Lundgren and Kiessling, 1985). Measurements of LDH activity in the pectoralis muscle of resident songbirds exposed to cold temperatures is lacking, but in migratory red knots (*Calidris canutus islandica*), LDH activity decreases with acclimation to low temperatures (Vézina et al., 2017). These findings suggest that the pectoralis muscle of small migratory songbirds is seasonally adjusted for sustained aerobic metabolism to support migratory flights, while reducing reliance on anaerobic pathways. In contrast, resident species do not alter their aerobic metabolism, which may be important for supporting thermoregulation (Marsh and Dawson, 1989; Swanson et al., 2020; Yacoe and Dawson, 1983). However, whether these changes in enzyme activity are the result of changes in muscle fiber type or flexibility of enzyme activity within a fiber type has not been investigated.

The objective of our study was to investigate whether resident and migratory sparrows exhibit seasonal plasticity in pectoralis muscle fiber type and enzyme activity that would be beneficial for supporting aerobically demanding seasonal activities. Songbirds (Order: Passeriformes) offer a valuable opportunity to explore trends of variation within families, given that various species within the same family can display a diverse range of migratory distances. In this study, we used Lincoln's sparrows [*Melopiza lincolnii* (Audubon 1834); Family: Passereillidae] which migrate between Northern Canada and the Southern USA, and song sparrows [*Melospiza melodia* (Wilson 1810); Family: Passereillidae], which migrate shorter distances between Southern Ontario, Canada and central USA (Kelly et al., 2019), as our migratory species. We compared these migrators to house sparrows [*Passer domesticus* (Linnaeus 1758); Family: Passeridae], which are resident in Southern Ontario year-round. Previous research indicates that CS activity in the pectoralis muscle of house sparrows does not change seasonally and that the muscle is composed entirely of FOG fibers (Lundgren and Kiessling, 1988; Swanson et al., 2020), but whether fiber type and enzyme activity are altered in migratory sparrows and/or with season has not been studied. Here, we aimed to test three hypotheses: (1) the seasonal hypothesis, that muscle morphology and enzyme activities change in response to daylight length changes; (2) the migratory hypothesis, that muscle morphology and enzyme activities will be dictated by migration distance; and (3) the fiber type hypothesis, that fiber type composition of the pectoralis muscle will dictate enzyme activity. To test these hypotheses, we stained for FOG and FG fiber types and measured CS and LDH activity in the pectoralis muscle of sparrows during short-day and long-day conditions.

## MATERIALS AND METHODS

### Birds and experimental design

Sparrows were caught at Long Point, Ontario, Canada, a stopover point during southbound migration, and at Western University, London, Ontario, Canada. Song sparrows (*Melospiza melodia*, *N*=16), Lincoln's sparrows (*Melospiza lincolnii*, *N*=6), and house sparrows (*Passer domesticus*, *N*=17) were caught at both locations between September and October 2021 and 2022 and were part of previous studies (Mohns et al., 2024). Sparrows in long daylight length (captured during September and October 2021) and house sparrows caught in February 2023 were a part of a large study encompassing measurements across various levels of tissue organization. Short daylight length individuals (captured during September and October 2022) were a part of a previous study assessing short daylight length on ventilatory responses to hypoxia (Mohns et al., 2024). All birds were housed at the Advanced Facility for Avian Research (London, Ontario, Canada) in individual cages (approximately 100×50×50 cm), fed *ad libitum* a seed mix diet, supplemented with mealworms (*Tenebrio molitor*) and had unlimited access to water. During September and October 2021 and 2022, birds were kept on a natural photoperiod (12.5 h light: 11.5 h dark), with a subset sampled under this regime as the ‘long-daylight’ photoperiod (12.5 h light: 11.5 h dark). The remaining sparrows then transitioned to a ‘short-daylight’ photoperiod (9 h light: 15 h dark) by mid-November to induce a non-migratory/wintering phenotype (Dick and Guglielmo, 2019; Reeves et al., 2003). Birds were kept on this photoperiod for ∼110 days before sampling. Nocturnal behavior was assessed for migratory species with audio and video recordings during the sampling periods to confirm that short-day birds were inactive at night and that long-day birds expressed migratory restlessness. A sub-set of house sparrows (*N*=4) was collected during February 2023 at Western University and they were sampled immediately after capture, resulting in a third treatment (short days with cold temperature; ∼10.5 h light: ∼13.5 h dark; daytime highs∼−1±2.1°C, night-time lows∼−11±4.4°C). Before terminal sampling, all animals were weighed. Animal capture and study procedures were approved by the University of Western Ontario Animal Care Committee (Protocols 2018-092, 2022-028) and Canadian Wildlife Service (SCOR-2018-0256, SCOR-2022-0256).

### Muscle histology

Pectoralis muscle samples were collected and prepared for histology and enzyme analysis at the time of sampling. All birds were humanely euthanized by decapitation under full isoflurane anaesthesia, and both pectoralis muscles were dissected out and weighed. Samples for enzyme activities were frozen in liquid N_2_ and stored at −80°C until assays were performed. For histology, pectoralis muscle was taken from the middle of the muscle, spanning from the subcutaneous surface to the sternum, as previously described (Ivy and Guglielmo, 2023). Samples were mounted on cork and coated in mounting medium (Cryomatrix; Thermo Fisher Scientific, Waltham, MA, USA), frozen in liquid N_2_-cooled isopentane, and stored at −80°C until sectioning. Samples were sectioned at 10 μm transverse to the muscle fiber length in a −20°C cryostat. Slices were then air-dried and stored at −80°C.

Muscle fiber types were determined by staining for myosin-ATPase activity to identify FOG and FG fibers, as has been previously used in other songbirds and geese (Chang et al., 2024; DuBay et al., 2020; Scott et al., 2009). Briefly, sections were brought to room temperature and preincubated in an acidic incubation solution (100 mmol l^−1^ sodium acetate, 10 mmol l^−1^ EDTA, pH 4.4) for 2 min. Slides were then rinsed in distilled (d) H_2_O and incubated in ATPase incubation buffer (200 mmol l^−1^ tris, 18 mmol l^−1^ CaCl_2_, 2.7 mmol l^−1^ ATP, pH 9.5) for 20 min with gentle agitation. Following a 15 min rinse in CaCl_2_ washing solution (1% w/v), slides were incubated in CoCl_2_ solution (2% w/v) for 10 min with gentle agitation. Slides were then rinsed in dH_2_O and developed in ammonium sulfide solution (2% w/v) for 30 s, rinsed in dH_2_O, and put through a dehydration sequence (95% ethanol, 100% ethanol, xylene) and mounted with Permount (Thermo Fisher Scientific, Waltham, MA, USA).

Capillaries were visualized by staining for alkaline phosphatase activity in a second set of pectoralis muscle sections, as has been previously used in geese (Scott et al., 2009). Briefly, sections were brought to room temperature and fixed in acetone for 5 min. After air drying for 10 min, slides were incubated with working buffer [1 mmol l^−1^ nitroblue tetrazolium (NBT) and 0.5 mmol l^−1^ 5-bromo-4-chloro-3-indoxyl phosphate, toluidine salt (BCIP), pH 9.3] for 1 h at 41°C. Slides were then rinsed in dH_2_O and mounted using Aquamount (Thermo Fisher Scientific, Waltham, MA, USA).

Sections were imaged using light microscopy on a Leica microscope (CTR6500) with Leica Application Suite imaging software. For both staining methods, stereological methods were used to make unbiased measurements (Egginton, 1990; Lui et al., 2015). Images were collected such that there was an equal representation across the entire muscle cross-section. Preliminary analysis indicated that between 12 and 16 images per section were sufficient to account for fiber type or capillary heterogeneity established by the number of images required to produce a stable mean value for each individual. All images were manually analyzed and counted for each fiber type and capillaries in ImageJ software (version 1.54i 03). The variables collected from each image for the muscle stain included: number of FOG and FG fibers, and transverse surface area of FOG and FG fibers, as has been described in birds (Ivy and Guglielmo, 2023; Schell et al., 2024; Scott et al., 2009). The variables collected from each image for the capillary stain included: number of capillaries and number of muscle fibers. These counts allowed for calculation of fiber density (number of FOG or FG fibers divided by image area), fiber numerical density (number of FOG or FG fibers relative to total fibers in each image), fiber areal density (total FOG or FG fiber transverse area divided by image area), capillary density (number of capillaries divided by image area), and capillary:fiber ratio (number of capillaries divided by number of fibers in an image) in each image.

### Enzyme activity assays

Pectoralis muscle tissue samples were homogenized for spectrophotometric enzyme assays of citrate synthase (CS) and lactate dehydrogenase (LDH) activities. Tissues were kept on ice and diluted in a 1:9 ratio of ice-cold homogenization buffer (20 mmol l^−1^ HEPES, 1 mmol l^−1^ EDTA, 0.1% Triton X-100). Samples were finely minced, then mechanically homogenized with low power for 10 s intervals with 30 s rests on ice in between (Dremel Multipro model 395, Racine, WI, USA). This was followed by 3 bouts of sonication for 10 s intervals separated by 30 s rests on ice (VirSonic 100, VirTis, Gardiner, NY, USA). The samples were centrifuged at 4°C, 10,000 ***g*** for 3 min (Centrifuge 5804 R, Eppendorf, Hamburg, Germany). The supernatant was used to measure the apparent maximal activity of CS and LDH at 39°C using a UV/Vis spectrophotometer with 1.5 ml cuvettes with a final volume of 1 ml (SpectraMax M2e, Molecular Devices, San Jose, CA, USA).

CS activity was assayed by measuring absorbance of 5,5′-dithiobis-2-nitrobenzoic acid (DTNB) at 412 nm, and LDH activity was assayed by measuring absorbance of NADH at 339 nm. Preliminary tests verified that substrate concentrations were saturating. The following assay conditions in mmol l^−1^ were used for each enzyme, with ‘±’ denoting the reagent omitted for background enzyme activity and respective buffers used to compensate for volume (Tris-HCl and imidazole). CS: ±1.5 oxaloacetate, 0.1 DTNB, 0.3 acetyl-CoA, 50 Tris-HCl (pH 8.0). A portion of the homogenate was further diluted in a 1:9 ratio for a final dilution of 1:99 for LDH assays. LDH: 0.15 NADH, ±1 pyruvate, 50 imidazole (pH 7.0). All enzyme assays were run in duplicate and corrected for background activity. Enzyme reaction rates were calculated using the extinction coefficients 14.15 mmol l^−1^ cm^−1^ (DTNB) and 6.22 mmol l^−1^ cm^−1^ (NADH).

### Statistical analysis

Bird mass and all pectoralis muscle histology variables and enzyme activities were analyzed using two-way ANOVAs to examine the main effects of species (house sparrow, song sparrow, Lincoln's sparrow), daylight length (‘long’ versus ‘short’ daylight), and their interaction. Holm–Šidák post-tests were used as appropriate. As pectoralis mass might be expected to covary with body mass, we used an ANCOVA with the same main effects and interactions as above plus body mass as a covariate. Owing to the addition of cold plus short-daylight house sparrows, one-way ANOVAs were conducted on all variables to examine the effects of daylight length (long-daylight, short-daylight and short-daylight plus cold conditions) within house sparrows. Holm–Šidák *post hoc* tests were performed as appropriate. An ANCOVA was performed on pectoralis mass with body mass as a covariate and daylight length as a main effect. We used linear models to assess whether pectoralis muscle fiber type composition influenced enzyme activity, with CS and LDH activities correlated against FOG and FG fiber densities. All values are reported as mean±s.e.m. and a significance level of *P*<0.05 was considered statistically significant. All statistical analyses were conducted with R Statistical Software (v. 4.2.0; r-project.org).

## RESULTS

### Body mass and pectoralis mass

**Fig. 1.**
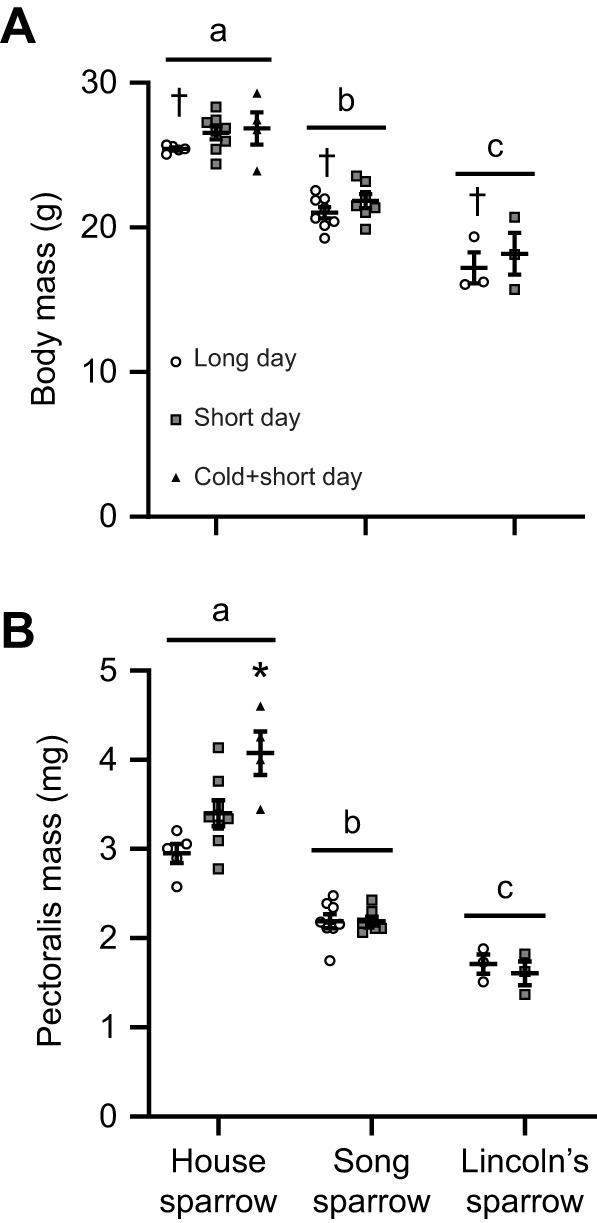
**Body mass and pectoralis mass in three species of sparrow under different photoperiod conditions.** Body mass (A) and pectoralis mass (B) differ significantly between house sparrows (*Passer domesticus*), song sparrows (*Melospiza melodia*) and Lincoln's sparrows (*Melospiza lincolnii*), with an overall increase in body mass in sparrows in short-day photoperiod conditions. Only house sparrows were sampled in cold plus short-day conditions, with these birds having a significantly heavier pectoralis compared with long- and short-day conditions. Individual data points are plotted along with means±s.e.m. Species that do not share a common letter are significantly different based on two-way ANOVA. ^†^Significant main effect of daylight length based on two-way ANOVA. *Significant difference between cold+short-day conditions and long- and short-day photoperiod based on one-way ANOVA within house sparrows. House sparrow: long-day, *N*=5; short-day; *N*=8; cold+short-day, *N*=4. Song sparrow: long-day, *N*=8; short-day; *N*=7. Lincoln's sparrow: long-day, *N*=3; short-day; *N*=3.

Body mass and pectoralis mass were significantly influenced by species and daylight length (Fig. 1). House sparrows had the largest body mass, being ∼22% larger than song sparrows and ∼47.5% larger than Lincoln's sparrows (main effect of species: *F*_2,28_=93.37, *P*<0.0001), while all species in short day length had overall larger body masses by ∼7.5% (main effect of day length: *F*_1,28_=4.240, *P*=0.005). Within house sparrows, body mass did not change with daylight length and/or cold (*F*_2,14_=1.459, *P*=0.266). Pectoralis mass mirrored body mass patterns (body mass was a significant covariate, *F*_1,27_=168.9, *P*<0.0001), with all species differing significantly (*F*_2,27_=6.797, *P*=0.004), such that house sparrow pectoralis mass was ∼47% larger than song sparrow and ∼95% larger than Lincoln's sparrow pectoralis mass (Fig. 1B). Pectoralis mass was not significantly influenced by daylight length (*F*_1,27_=0.965, *P*=0.335) and there was a trend for an interaction between species and daylight length (*F*_2,27_=3.105, *P*=0.061). However, house sparrows in the cold plus short-daylight treatment had significantly larger pectoralis muscle mass (*F*_2,13_=7.199, *P*=0.008); ∼38% larger compared with house sparrows at long-daylight and ∼20% compared with short-daylight conditions (body mass covariate, *F*_1,13_=14.55, *P*=0.002).

### Pectoralis histology

**Fig. 2.**
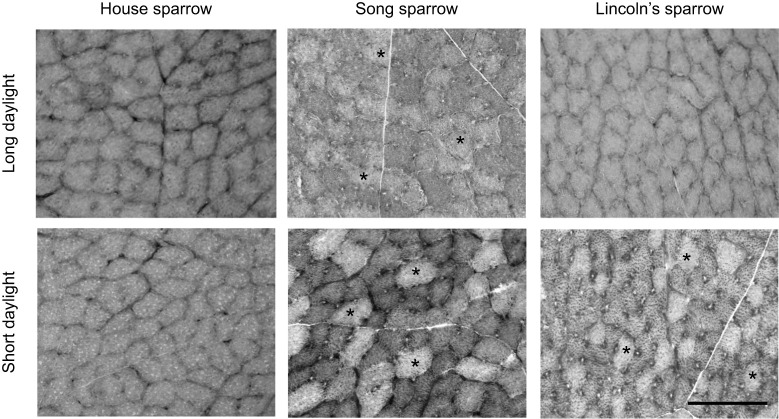
**Representative images of the pectoralis muscle of the house sparrow, song sparrow and Lincoln's sparrow during long- and short-daylight conditions.** Myosin ATPase staining was used to identify fast-oxidative glycolytic fibers and fast-glycolytic fibers (asterisks identify some fast-glycolytic fibers, but not all in each image). Images are in grayscale to improve visualization. Scale bar: 100 μm.

**Fig. 3.**
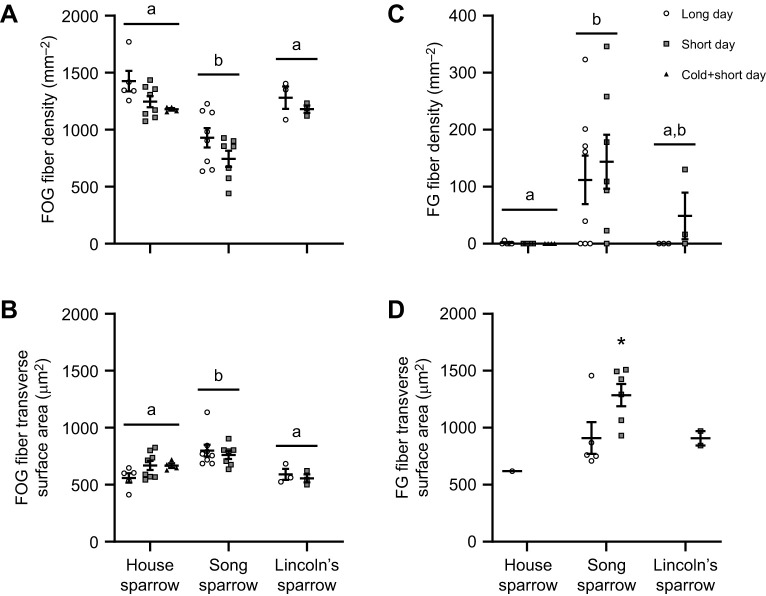
**Fiber density and transverse surface area of fast-oxidative glycolytic (FOG) and fast glycolytic (FG) fibers in house sparrows.** Fiber density (number of FOG or FG fibers relative to muscle surface area; A,C) and transverse surface area (B,D) of FOG and FG fibers in house sparrows, song sparrows and Lincoln's sparrows. Song sparrows had a significantly lower FOG fiber density, with larger transverse surfaces areas, and had FG fibers present regardless of photoperiod condition. Lincoln's sparrows only incorporated FG fibers during short-day photoperiod conditions, while house sparrows did not alter fiber type. Individual data points are plotted along with means±s.e.m. Species that do not share a common letter are significantly different based on two-way ANOVA. *Significant difference between long- and short-daylight conditions within a species. House sparrow: long-day, *N*=5; short-day; *N*=8; cold+short-day, *N*=4. Song sparrow: long-day, *N*=8; short-day; *N*=7. Lincoln's sparrow: long-day, *N*=3; short-day; *N*=3.

FOG fiber density, numerical density, transverse surface area, and areal density were influenced by species and daylight length (Figs 2 and 3, Table 1). FOG fiber density was significantly influenced by species (*F*_2,28_=24.32, *P*<0.0001), with song sparrows having a significantly lower FOG fiber density overall compared to house sparrows and Lincoln sparrows (Fig. 3A). FOG fiber density was also influenced by a main effect of daylight length (*F*_1,28_=6.759, *P*=0.015), although there was no significant interaction between species and daylight length, with sparrows in short-day conditions having overall lower FOG fiber densities. The addition of the cold plus short-daylight treatment in house sparrows resulted in a significant treatment effect (*F*_2,14_=3.720, *P*=0.050), although no significant difference between groups was observed with *post hoc* testing. FOG numerical density was also significantly lower in song sparrows (main effect of species: *F*_2,28_=8.193, *P*=0.0016), but was not influenced by daylight length (main effect of daylight length: *F*_1,28_=0.621, *P*=0.437; Table 1), nor within house sparrows (main effect of treatment: *F*_2,14_=1.235, *P*=0.321). FOG fiber transverse area was significantly larger in song sparrows compared with house sparrows and Lincoln's sparrows (main effect of species: *F*_2,28_=10.35, *P*=0.0004), but was not influenced by daylight length (*F*_1,28_=0.195, *P*=0.662; Fig. 3B). FOG fiber areal density was also influenced by species (*F*_2,28_=6.271, *P*=0.006; Table 1), with song sparrows having lower areal density compared with house sparrows but not Lincoln's sparrows, with a trend for short-daylight sparrows having lower FOG areal density compared with long-daylight sparrows (*F*_1,28_=3.797, *P*=0.061; Species×Daylight length interaction: *F*_2,28_=2.444, *P*=0.105; Table 1). The inclusion of a cold plus short-daylight treatment on house sparrows did not significantly alter FOG fiber transverse surface area (*F*_2,14_=2.271, *P*=0.140) or areal density (*F*_2,14_=0.710, *P*=0.509).

**Table 1.** Numerical density and areal density of fast-oxidative glycolytic (FOG) and fast glycolytic (FG) fibers in pectoralis muscle of house sparrow (*Passer domesticus*), song sparrow (*Melospiza melodia*) and Lincoln's sparrow (*Melospiza lincolnii*)

	*N*	Numerical density (%)		Areal density (%)	
		FOG	FG	FOG	FG
House sparrow					
Long daylight	5	99.9±0.1	0.09±0.1	77.7±2.4	0.07±1.0
Short daylight	8	100	0	81.0±2.1	0
Cold+short daylight	4	100	0	78.0±2.0	0
Song sparrow					
Long daylight	8	87.2±5.0	12.8±5.0	72.0±6.0	9.06±3.4
Short daylight	7	82.4±6.1	17.6±6.1	55.8±5.4	16.6±4.6
Lincoln's sparrow					
Long daylight	3	100	0	74.2±2.0	0
Short daylight	3	96.1±3.3	3.9±3.3	64.7±1.8	4.1±3.4

Values are mean±s.e.m. numerical density (number of FOG or FG fibers relative to total number of fibers) and areal density (total area of FOG or FG fibers relative to image area).

FG fibers were identified in song sparrows and Lincoln's sparrows, with only 1 house sparrow having FG fibers present (Figs 2 and 3, Table 1). Song sparrows had FG fibers in both long and short daylight conditions, although not all individuals had FG present (5 out of 8 and 6 out of 7, in long and short daylight, respectively; Fig. 3C). In Lincoln's sparrows, FG fibers were only present during short-daylight conditions, and 2 out of 3 individuals had FG fibers present. Only 1 (out of 5) house sparrow in long daylight conditions had FG fibers present (Fig. 3C). FG density was significantly influenced by a main effect of species (*F*_2,28_=8.229, *P*=0.002), but not daylight length (*F*_1,28_=0.579, *P*=0.453). FG fiber numerical density was significantly higher in song sparrows compared with house sparrows and Lincoln's sparrows (main effect of species: *F*_2,28_=8.193, *P*=0.002), but was not influenced by daylight length (*F*_1,28_=0.621, *P*=0.437; Table 1). Song sparrow FG transverse surface area was larger during short-daylight conditions (*P*=0.039), with FG fiber areal density significantly larger in song sparrows compared with house sparrows and Lincoln's sparrows (main effect of species: *F*_2,28_=10.36, *P*=0.0004; main effect of season: *F*_1,24_=2.405, *P*=0.132; Table 1).

**Fig. 4.**
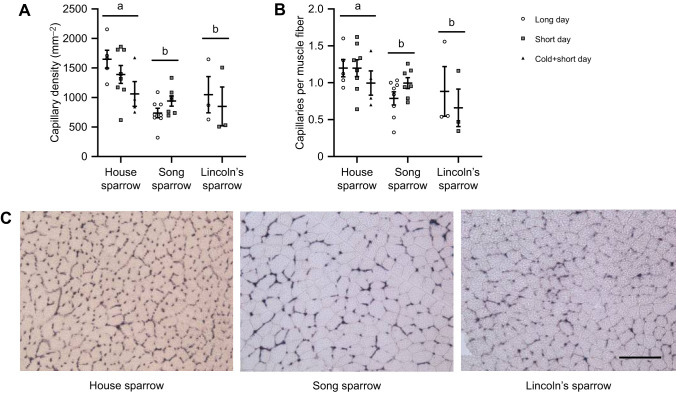
**Capillary density and capillary-fiber ratio in the pectoralis muscle of three species of sparrow.** Capillary density (number of capillaries relative to muscle surface area; A) and capillary-fiber ratio (B) are significantly greater in house sparrows compared with song sparrows and Lincoln's sparrows, but are not affected by photoperiod. (C) Representative images of capillary staining using alkaline phosphatase in the pectoralis muscle for each species during long-daylight conditions. Individual data points are plotted along with means±s.e.m. Species that do not share a common letter are significantly different based on two-way ANOVA. House sparrow: long-day, *N*=5; short-day; *N*=8; cold+short-day, *N*=4. Song sparrow: long-day, *N*=8; short-day; *N*=7. Lincoln's sparrow: long-day, *N*=3; short-day; *N*=3. Scale bar: 100 μm.

Capillary measurements were significantly influenced by species, but not daylight length (Fig. 4). Capillary density was significantly lower in song sparrows and Lincoln's sparrows compared with house sparrows (*F*_2,28_=12.25, *P*=0.0002), but was not altered with daylight length (*F*_1,28_=0.105, *P*=0.749; Fig. 4A). Capillary:fiber ratio was also lower in song sparrows and Lincoln's sparrows compared with house sparrows (*F*_2,28_=5.167, *P*=0.012), but was not influenced by daylight length (*F*_1,28_=0.236, *P*=0.631; Fig. 4B). Within house sparrows, capillary density and capillary:fiber ratio were not influenced by treatment (*F*_2,14_=2.354, *P*=0.131; *F*_2,14_=0.655, *P*=0.535, respectively).

### Pectoralis muscle enzyme activities

**Fig. 5.**
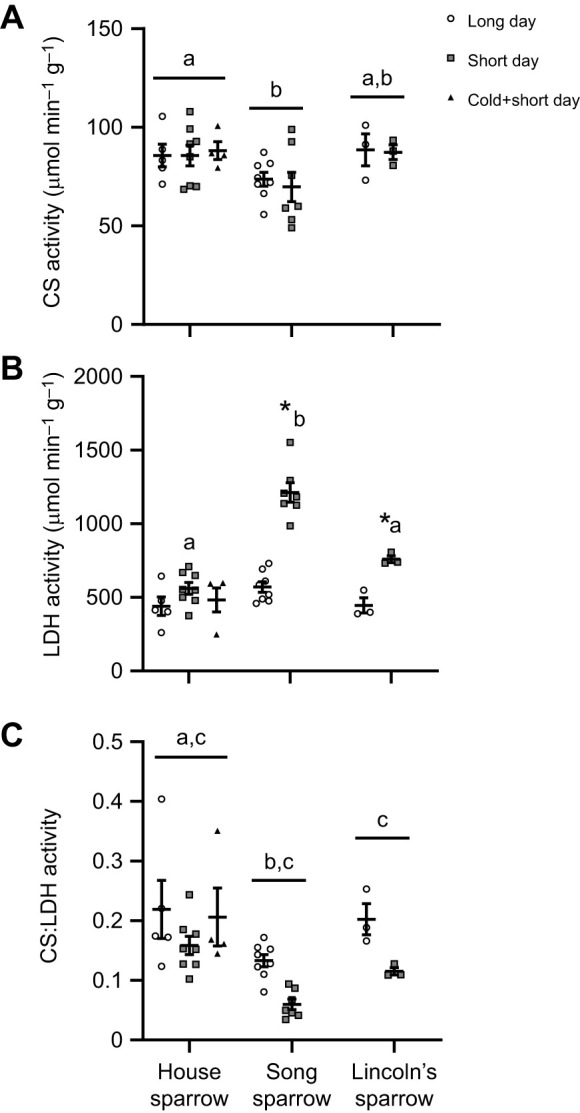
**Effect of photoperiod on citrate synthase and lactate dehydrogenase activity in three sparrow species.** Citrate synthase activity (CS, A) is not influenced by photoperiod, while lactate dehydrogenase activity (LDH, B) and the ratio of CS:LDH are significantly influenced by sparrow species and/or short-day photoperiod. Song sparrows and Lincoln's sparrows increased LDH activity with short-day photoperiod, while house sparrows exhibited no changes with photoperiod. Individual data points are plotted along with means±s.e.m. Species that do not share a common letter are significantly different based on two-way ANOVA. *Significant difference between long- and short-day photoperiod based on pairwise post-tests. House sparrow: long-day, *N*=5; short-day; *N*=8; cold+short-day, *N*=4. Song sparrow: long-day, *N*=8; short-day; *N*=7. Lincoln's sparrow: long-day, *N*=3; short-day; *N*=3.

Citrate synthase activity and lactate dehydrogenase activity differed significantly among species, but only LDH activity was influenced by daylight length (Fig. 5). Song sparrows had lower CS activity compared with house sparrows, but not Lincoln's sparrows (main effect of species: *F*_2,28_=4.409, *P*=0.022; Fig. 5A). Daylight length did not affect CS activity (*F*_1,28_=0.825, *P*=0.372; Fig. 5A). The addition of house sparrows in cold plus short daylight also did not change CS activity (main effect of treatment: *F*_2,14_=0.055, *P*=0.947). In contrast, we found a significant interaction between species and daylight length with lactate dehydrogenase activity (*F*_2,28_=14.80, *P*<0.0001; Fig. 5B). Song sparrows and Lincoln's sparrows significantly increased LDH activity in short-daylight compared with long-daylight conditions, by ∼113% and ∼70% respectively, but not in house sparrows. LDH activity was also higher in song sparrows in short-daylight conditions compared with house sparrows and Lincoln's sparrows, by ∼116% and ∼60% respectively. LDH activity was similar among all sparrow species in long-daylight conditions (main effect of species: *F*_2,28_=29.44, *P*<0.0001; main effect of daylight length: *F*_1,28_=79.71, *P*<0.0001). In house sparrows, the addition of cold plus short-daylight treatment did not affect LDH activity (*F*_2,14_=1.535, *P*=0.250). Sparrow species had a significant effect on the CS:LDH ratio (*F*_2,28_=9.427, *P*=0.0007), with song sparrows having a similar ratio to Lincoln's sparrows but a significantly lower ratio than house sparrows. CS:LDH activity was also influenced by a main effect of daylight length (*F*_1,28_=15.72, *P*=0.0005), with a lower ratio during short-daylight conditions. No differences were observed between treatments with the addition of cold plus short-daylight treatment within house sparrows for CS:LDH ratio (*F*_2,14_=1.025, *P*=0.0.384).

**Fig. 6.**
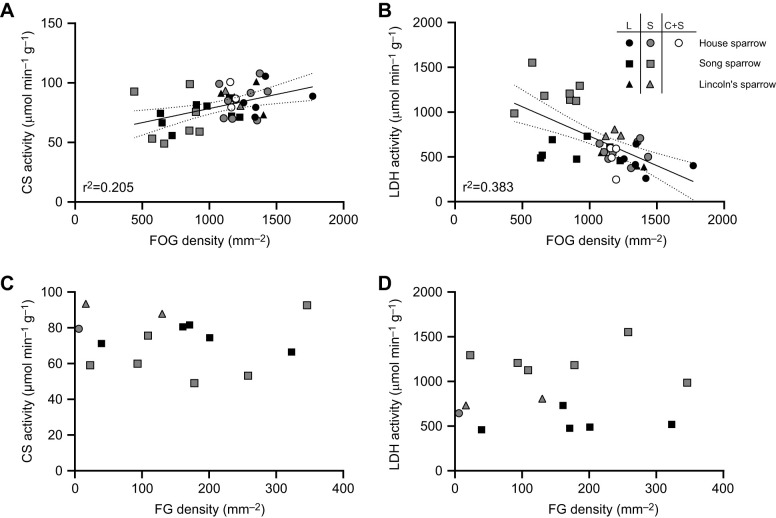
**Effect of muscle fiber type on citrate synthase and lactate dehydrogenase activity in three sparrow species.** Citrate synthase (CS) activity is positively correlated with fast-oxidative glycolytic (FOG) fiber density (*R*^2^=0.205, A) but not fast glycolytic (FG) fiber density (*R*^2^=0.004, C). Lactate dehydrogenase (LDH) activity is negative correlated with FOG density (*R*^2^=0.383, B) but not FG density (*R*^2^=0.003, D). Individuals with no FG fibers present were removed from correlations with FG density. Individual points are plotted with a line of best fit and 95% confidence interval where appropriate. House sparrow: long-day, *N*=5; short-day; *N*=8; cold+short-day, *N*=4. Song sparrow: long-day, *N*=8; short-day; *N*=7. Lincoln's sparrow: long-day, *N*=3; short-day; *N*=3. L, long-day; S, short-day; C+S, cold and short-daylight treatments.

Lactate dehydrogenase and citrate synthase activity correlated with muscle fiber type density (Fig. 6). Citrate synthase exhibited a positive correlation with FOG fiber density (*R* ^2^=0.205, *P*=0.007; Fig. 6A), but not FG fiber density (*P*=0.835). Similarly, LDH activity negatively correlated with FOG fiber density (*R*^2^=0.383, *P*<0.0001; Fig. 6B), but was unrelated to FG fiber density (*P*=0.844).

## DISCUSSION

The pectoralis muscle in birds undergoes seasonal changes, but whether this flexibility is the result of changes in muscle fiber type or depends on a species' migratory strategy has not been well explored. Additionally, pectoralis enzyme activities exhibit seasonal flexibility, but whether these changes are associated with changes in fiber type (the fiber-type hypothesis) is not known. We investigated whether muscle fiber type and enzyme activities were altered with changes in daylight length (the seasonal hypothesis) in two migratory sparrow species, which rely on the pectoralis muscle for migratory flight, and one resident sparrow species (the migratory hypothesis), which relies on the pectoralis muscle for thermoregulation during winter. We found that migratory sparrows had FG fibers present in their pectoralis muscle, with Lincoln's sparrows incorporating more FG fibers during the short-daylight condition, while no FG fibers were present in the resident house sparrows. Citrate synthase activity was not influenced by daylight length but was lower in one migratory sparrow species compared with resident sparrows. By contrast lactate dehydrogenase activity significantly increased during short daylight in the migratory sparrows, but not in the resident sparrow. These findings suggest that the presence of FG fibers can facilitate increases in pectoralis LDH activity during short-daylight conditions. Additionally, resident sparrows do not exhibit seasonal plasticity in fiber type or enzyme activities, suggesting that FOG fibers are capable of supporting all daily activities throughout the year, including thermoregulation.

### The migratory hypothesis

We observed FG fibers and lower capillary density in the migratory sparrow species but not in the resident sparrow species. We predicted that migratory species would have a lower proportion of FG fibers and higher aerobic enzyme activity compared with resident species. Song sparrows and Lincoln's sparrows had FG fibers, providing further evidence that small songbirds can have FG fibers (DuBay et al., 2020; Lundgren and Kiessling, 1988; Rosser and George, 1986). Song sparrows did have a greater density of FG fibers during long-daylight conditions compared with Lincoln's sparrows, which partially supports our migration prediction, as song sparrows generally migrate shorter distances than Lincoln's sparrows (Kelly et al., 2019). FG fiber transverse surface area was similar to FOG fibers in song sparrows and Lincoln's sparrows, unlike in geese and waterfowl, where FG fibers have higher transverse surface areas (Parr et al., 2021). It is possible that, in our species, FOG fibers were transitioning into FG fibers (Plotkin et al., 2021) and that they had not reached their final size even after ∼110 days in captivity. However, we acknowledge that ∼110 days in captivity with lower levels of flight activity compared with that of birds in the wild could impact muscle structure and function, and thus our observed findings. In contrast to our seasonal prediction, house sparrows did not have any FG fibers, which agrees with previous findings (Rosser and George, 1986), although the season in which their sample was taken was not reported. We also found that song sparrows and Lincoln's sparrows had lower capillary density and capillary:fiber ratio compared with house sparrows, which supports the lower number of capillaries typically found around FG fibers.

Why might song sparrows and Lincoln's sparrows have FG fibers? FG fibers can be important for sudden maneuvers that require rapid acceleration, such as burst flight (Parker and George, 1975), associated with ground foraging. In fact, many North American ground-foraging birds have FG fibers in the pectoralis, including American robins (*Turdus migratorius*), white-throated sparrows (*Zonotrichia albiocollis*), common grackles (*Quiscalus quiscula*) and cowbirds (*Molothrus ater*) (Rosser and George, 1986). In contrast, ground-foraging dark-eyed juncos (*Junco hyemalis*), which have a similar migratory range to song sparrows and would have similar thermoregulatory challenges, do not have pectoralis FG fibers (Rosser and George, 1986). These differences in pectoralis muscle fiber type composition within a family and among families may suggest that foraging behavior may interact with migratory and thermoregulatory requirements, in addition to many other factors, making it difficult to parse out why some small songbirds have FG fibers.

Song sparrows had a lower density of FOG fibers, but overall larger FOG fiber transverse surface areas compared with Lincoln's sparrows and house sparrows. The larger transverse surface area of FOG fibers in song sparrows resulted in no difference in FOG areal density between species (Fig. 2, Table 1). This larger transverse surface area may be important for maintaining aerobic capacity and power generation to support flight (Greenewalt, 1962; Jimenez, 2020), despite the lower overall CS activity we observed in this species. FOG fibers would also be important for maintaining thermoregulation in cold climates, which song sparrows could experience, despite migrating (Kelly et al., 2019), suggesting that song sparrows may have evolved larger FOG transverse surface areas to support the aerobic capacity needed for thermoregulation and migratory flight while also having FG fibers.

Pectoralis muscle enzyme activity exhibited seasonal flexibility only in the migratory sparrow and not the resident sparrow species. CS activity was lower in song sparrows compared with house sparrows, but not Lincoln's sparrows, in contrast to our predictions. However, LDH activity did exhibit seasonal plasticity but only in song sparrows and Lincoln's sparrows, suggesting that migratory species may be better able to adjust their anaerobic enzyme activity compared with resident species. These findings suggest that migratory sparrow species may alter anaerobic enzyme activity according to their energetic requirements, while this adjustment may be too costly and/or not necessary for resident species.

### The seasonal hypotheses

Daylight length influences pectoralis muscle fiber type and anaerobic enzymes in migratory sparrow species, but not the resident sparrow species. For the seasonal hypothesis, we predicted that FOG fiber density and aerobic enzyme activities would be highest during long-daylight conditions in migratory species, when aerobic requirements are greatest, and highest during short-daylight conditions in resident species, when aerobic requirements are greatest owing to thermoregulation. Our prediction was partially supported in Lincoln's sparrows, which only had FOG fibers during long-daylight conditions and incorporated FG fibers only during short-daylight conditions, similar to previous finding in other short-distance migratory songbirds (Chang et al., 2024). We observed no changes in fiber type in song sparrows, but song sparrows and Lincoln's sparrows increased LDH activity during short-daylight conditions, similar to previous measurements in migratory songbirds (Dick, 2017). In contrast to our predictions and previous findings in migratory songbirds (Coulson et al., 2024; Dick, 2017; Lundgren and Kiessling, 1985, 1986), we observed no changes in CS activity with daylight length, similar to findings in resident songbird species (Marsh and Dawson, 1989; Swanson et al., 2020; Yacoe and Dawson, 1983). The lack of change in CS activity could be important for supporting thermoregulation in song sparrows, as they experience cold climates in their non-breeding season (Kelly et al., 2019), while increases in LDH activity and FG fibers could suggest that these species are spending more time on the ground foraging and/or conducting burst/escape flights from predators.

House sparrows did not change fiber type or enzyme activities seasonally. In this study we had an additional treatment of cold plus short daylight with our freshly caught house sparrows. This treatment allowed us to investigate whether acclimatization to cold temperatures may further pronounce changes in the muscle fiber type and enzyme activities. We found that cold plus short-daylight conditions resulted in a significantly larger pectoralis mass compared with the long- and short-daylight treatments, and that this was possibly associated with hypertrophy of the FOG fibers. Unfortunately, our findings only trended towards statistical significance, suggesting that a larger sample size may be required to test this hypothesis. A change in FOG fiber density and transverse surface area would be important for fueling thermogenesis (Jimenez, 2020) and similar findings have been reported in other small songbirds in cold climates (Liknes and Swanson, 2011; Swanson, 2010; Swanson and Merkord, 2013). Moreover, neither CS nor LDH activities changed, consistent with findings in resident songbirds (Marsh and Dawson, 1989; Swanson et al., 2020; Yacoe and Dawson, 1983), suggesting that a maintenance of CS and LDH is important regardless of season.

It is important to note that a lack of change in muscle fiber type does not necessarily imply a lack of changes in myosin heavy chain (MHC) isoforms. Previous research on white-crowned sparrows (*Zonotrichia leucophrys gambelli*) showed that although the pectoralis muscle comprises only FOG fibers, MHC isoforms changed seasonally (Velten et al., 2016). The lack of changes in house sparrow fiber type between seasons could mean that changes in MHC isoforms are able to support the seasonal changes in muscle mechanical output instead of requiring changes in fiber type. Whether song sparrows and Lincoln's sparrows also exhibit changes in MHC isoforms between seasons in addition to changes in fiber type is unknown. Further research into the seasonal plasticity of MHC isoforms in songbird flight muscle is required.

### The fiber-type hypothesis

Lastly, we investigated whether pectoralis muscle fiber composition would dictate enzyme activity, as previous studies demonstrating seasonal flexibility in enzyme activities have not considered muscle fiber type. Given that house sparrows and song sparrows did not exhibit flexibility in muscle fiber type, but that song sparrows and Lincoln's sparrows did exhibit season flexibility in LDH activity, our findings suggest that the presence of FG fibers are important for flexibility in LDH activity. This relationship between FG fibers and LDH activity may only be true for sparrows and small songbirds, as yellow-rumped warblers, *Setophaga coronata*, increase LDH activity during the non-migratory period (Dick, 2017) and incorporate FG fibers (Chang et al., 2024). Additionally, we observed no changes in CS activity with daylight length, despite a main effect of daylight length reducing FOG density overall. We found that LDH activity was negatively associated with FOG fiber density and that CS activity was positively correlated with FOG fiber density, but there was no relationship with LDH and CS activity with FG fiber density. This could suggest that the lower FOG fiber density (and presumed larger transverse surface area, although there was only a trend with our data) may be important for identifying fibers that will transition into FG fiber phenotype.

In conclusion, we show that FG fibers are present in two more small migratory songbird species, and that the pectoralis muscle of migratory sparrows can exhibit seasonal flexibility in fiber type and enzyme activity. FG fibers were present in song sparrows regardless of daylight length, while Lincoln's sparrows integrated FG fibers during the short daylight length conditions. Neither migratory species altered CS enzyme activity, but both exhibited seasonal flexibility in LDH activity, with higher activity during short daylight length. In contrast, house sparrows did not alter muscle fiber type or CS and LDH activities. Our findings suggest that seasonal flexibility in the pectoralis muscle is important for migratory species, whereas in a resident sparrow species consistency in fiber type, CS and LDH activity are important across seasons.
